# Distortion correction of echo‐planar diffusion‐weighted images of uterine cervix

**DOI:** 10.1002/jmri.25080

**Published:** 2015-10-20

**Authors:** Nandita M. deSouza, Matthew Orton, Kate Downey, Veronica A. Morgan, David J. Collins, Sharon L. Giles, Geoffrey S. Payne

**Affiliations:** ^1^ CRUK/EPSRC Cancer Imaging CentreInstitute of Cancer Research and Royal Marsden NHS Foundation TrustSuttonSurreyUK

**Keywords:** diffusion‐weighted, distortion correction, reverse gradient, cervix cancer

## Abstract

**Purpose:**

To investigate the clinical utility of the reverse gradient algorithm in correcting distortions in diffusion‐weighted images of the cervix and for increasing diagnostic performance.

**Materials and Methods:**

Forty‐one patients ages 25–72 years (mean 40 ± 11 years) with suspected or early stage cervical cancer were imaged at 3T using an endovaginal coil. *T*_2_‐weighted (W) and diffusion‐weighted images with right and left phase‐encode gradient directions were obtained coronal to the cervix (b = 0, 100, 300, 500, 800 s mm^−2^). Differences in angle of the endocervical canal to the x‐axis between *T*_2_W and right‐gradient, left‐gradient, and corrected images were measured. Uncorrected and corrected images were assessed for diagnostic performance when viewed together with *T*_2_W images by two independent observers against subsequent histology.

**Results:**

The angles of the endocervical canal relative to the x‐axis were significantly different between the *T*_2_W images and the right‐gradient images (*P* = 0.007), approached significance for left‐gradient images (*P* = 0.055), and were not significantly different after correction (*P* = 0.95). Corrected images enabled a definitive diagnosis in 34% (*n* = 14) of patients classified as equivocal on uncorrected images. Tumor volume in this subset was 0.18 ± 0.44 cm^3^ (mean ± SD; sensitivity of detection 100% [8/8], specificity 50% [3/6] for an experienced observer). Correction did not improve diagnostic performance for the less‐experienced observer.

**Conclusion:**

Distortion‐corrected diffusion‐weighted images improved correspondence with *T*_2_W images and diagnostic performance in a third of cases. J. Magn. Reson. Imaging 2016;43:1218–1223.

Cervical cancer accounts for 1 in 10 cancers diagnosed in women worldwide.^1^ Because of its soft‐tissue contrast, magnetic resonance imaging (MRI) is the modality of choice employed in diagnosis and staging of cervical cancer.^2^ Diffusion‐weighted MRI (DW‐MRI) has been employed to further improve the detection of small cervical tumors because of the significantly lower apparent diffusion coefficient (ADC) of cervical tumor compared to normal cervical tissue.^3^

Diffusion‐weighted images suffer from distortion induced by susceptibility variation and severe eddy‐currents. Eddy‐current‐related distortions are caused by strong diffusion‐sensitizing magnetic field gradients that have short ramp times and they depend on the magnitude and direction of the diffusion‐encoding field gradients. This adds to the susceptibility induced distortions in echo planar imaging (EPI)‐based sequences where residual eddy‐currents cause translation (B_0_ eddy‐current field), shear (frequency‐encode direction eddy‐current gradient field) and scaling (phase‐encode direction eddy‐current gradient field).^4^, ^5^ These combined image distortions are worst in the phase‐encode direction and spatial distortions along the phase‐encoding axis are several orders of magnitude larger than those along the frequency‐encoding axis.

Several approaches have been proposed to correct for spatial distortions on MR images. Most of these methods were devised to correct for EPI‐based distortions and were later extended to DW‐EPI‐based sequences. The reverse gradient technique was originally proposed by Chang and Fitzpatrick to correct for susceptibility‐induced artifacts in standard spin echo images.^6^ The technique requires the acquisition of two images of the same object under the same conditions except for the polarity of the frequency‐encode line. This relies on the fact that if a second acquisition is performed under the same conditions except for the polarity of the frequency‐encode gradient, then the spatial shifting of the signal in the second image will occur in the opposite direction. The pair of 2D images can be treated as a collection of independent pairs of 1D images, one for each value of the frequency‐encode line.

Initial pilot data reported in a conference proceedings^7^ indicated the technical potential of using the reverse gradient technique in the phase‐encode direction for assessing the uterine cervix. In the present study we investigated the clinical utility of the technique in correcting distortions in DW images of the cervix and for increasing diagnostic performance.

## Patients and Methods

### Image Acquisition

A total of 41 patients age 25–72 years, (mean 40 ± 11 years) with cervical cancer were prospectively recruited for this protocol and underwent endovaginal MRI with written informed consent under a protocol approved by our institutional research ethics committee. Patients were studied with an empty bladder and following 20 mg of hyoscine butylbromide administered intramuscularly to reduce bowel peristalsis.

Imaging was performed on a 3.0T Philips Achieva MR system (Philips Healthcare, Best, The Netherlands). The endovaginal receiver coil was developed in‐house.^3^ A turbo spin‐echo (SE) sequence was used to acquire *T*_2_‐weighted (*T*_2_W) images in three planes orthogonal to the cervix. The chosen parameters were a field‐of‐view (FOV) of 100 mm, echo time 80 msec, repetition time 3400 msec, spectral presaturation inversion recovery fat suppression (SPIR), left–right phase encoding, two averages, acquisition matrix image matrix 288 × 288, 0.35 mm resolution, 24 slices with 2 mm slice thickness and 0.2 mm separation. *T*_2_W images were used to plan DWIs that were acquired coronal to the cervix using a single‐shot SE EPI‐based sequence. In this sequence, a non‐coplanar application of a π/2 and π pulse reduces the SE selection domain in the phase‐encoding direction without incurring aliasing artifacts.^7^ The chosen parameters were an FOV of 100 mm, echo time 52 msec, repetition time 8000 msec, SPIR fat suppression, left–right phase encoding, one average, bandwidth 9.6 Hz/pixel, EPI factor 115 acquisition matrix 80 × 80, 1.25 mm in‐plane resolution, image reconstruction matrix 224 × 224, *b*‐values of 0, 100, 300, 500, and 800 s mm^−2^, 24 slices with 2 mm slice thickness and 0.2 mm separation. A left–right phase encoding was chosen as opposed to anterior–posterior encoding to avoid artifacts through the cervix. The long repetition time in the diffusion sequence was due to the extra outer volume suppression pulses added to improve the quality of the acquired images. The gradient reversal method was applied along the phase‐encode (left–right) axis as distortions along the phase‐encode axis are several orders of magnitude larger than those along the frequency‐encode axis for single‐shot EPI‐based sequences. Acquisition time for the DW images was 4 minutes 33 seconds for each gradient direction.

### Image Processing and Analysis

Diffusion images were resized to the scale of the *T*_2_W images using a sinc interpolant. In the computation of reverse gradient correction all *b*‐values of 0, 100, 300, 500, and 800 s mm^−2^ images were considered. No diffusion sensitization gradients were applied in the acquisition of the *b* = 0 s mm^−2^ images and the largest diffusion gradients were applied in the acquisition of the *b* = 800 s mm^−2^ (compared with other b values). The utility of the *b* = 0 s mm^−2^ image was that it has the highest signal‐to‐noise ratio (SNR), while the *b* = 800 s mm^−2^ image has the lowest SNR but highlights abnormality within tissue more clearly due to diffusion restriction. The corrected images were generated from the combination of both distorted right and left gradient images. Apparent diffusion coefficient (ADC) maps were then computed for the corrected diffusion images. The Chang–Fitzpatrick algorithm was implemented in MatLab (MathWorks, Natick, MA) using the approach described previously.^6^ Specifically, the integral form of the rectification equation (eq. 13 in Ref. 6) was computed using a direct discrete approximation, and this was combined with Lanczos interpolation (a normalized sinc function multiplied by a sinc window) of the cumulative image values to determine the undistorted position.

To assess the correction algorithm, the angles between the endocervical canal (a line along its long axis from ectocervix to internal os) and the x‐axis on the *T*_2_W, left gradient, right gradient, and corrected DW images were measured by an observer with 20 years (N.d.S.) experience of endovaginal MRI. The largest diffusion gradient *b* = 800 s mm^−2^ images were chosen for the angle measurement on the images, as they exhibited the largest visual distortion. This provided an objective measure of the similarity of the *T*_2_W ground truth image with each of the right gradient, left gradient, and corrected DW images. In addition, template matching utilizing normalized cross‐correlation (NCC)^11^ for *b* = 800 s mm^−2^ images was computed for three slices through the center of the cervix of each patient, as this captured most anatomic features. For these slices, each of the right gradient, left gradient, and the corrected diffusion images was matched to the corresponding *T*_2_W image, with a kernel k of size 25 × 25 pixels (8.8 mm × 8.8 mm) and a search neighborhood t of 51 × 51 pixels (18 mm × 18 mm). An NCC map was obtained for each pair of *b* = 800 s mm^−2^ and *T*_2_W images.

### Diagnostic Performance

In addition, the correction was assessed qualitatively for improvement in the diagnostic performance by two independent observers of 20 years (N.d.S.) and 3 years (K.D.) experience of endovaginal MRI. Images were scored as positive, negative, or equivocal for tumor on viewing the *T*_2_W and left gradient images together, and the *T*_2_W and right gradient images together. In the equivocal group, the *T*_2_W+corrected images were viewed together and scored either as positive or negative for tumor. Sensitivity and specificity for detecting tumor were calculated against subsequent histology in 35 cases, using cone or LLETZ biopsy (*n* = 10) or surgical resection (trachelectomy *n* = 9, hysterectomy *n* = 16). Six cases had prior punch biopsies positive for tumor, and tumor volume on imaging dictated their subsequent management with chemoradiation.

All cases that were scored as positive for tumor by the experienced observer had tumor volume measured on the *T*_2_W images by drawing a region of interest (ROI) around an intermediate signal intensity lesion that showed corresponding diffusion restriction and multiplying the sum of the areas by the slice thickness.

### Statistical Analysis

Results are quoted as mean ± standard deviation (SD). Differences between the angle of the endocervical canal with the horizontal on right gradient, left gradient, and corrected images were compared using a paired *t*‐test (Excel for Windows) and a value <0.05 used to denote significance. Interobserver agreement was assessed using Cohen's kappa.

## Results

**Figure 1:**
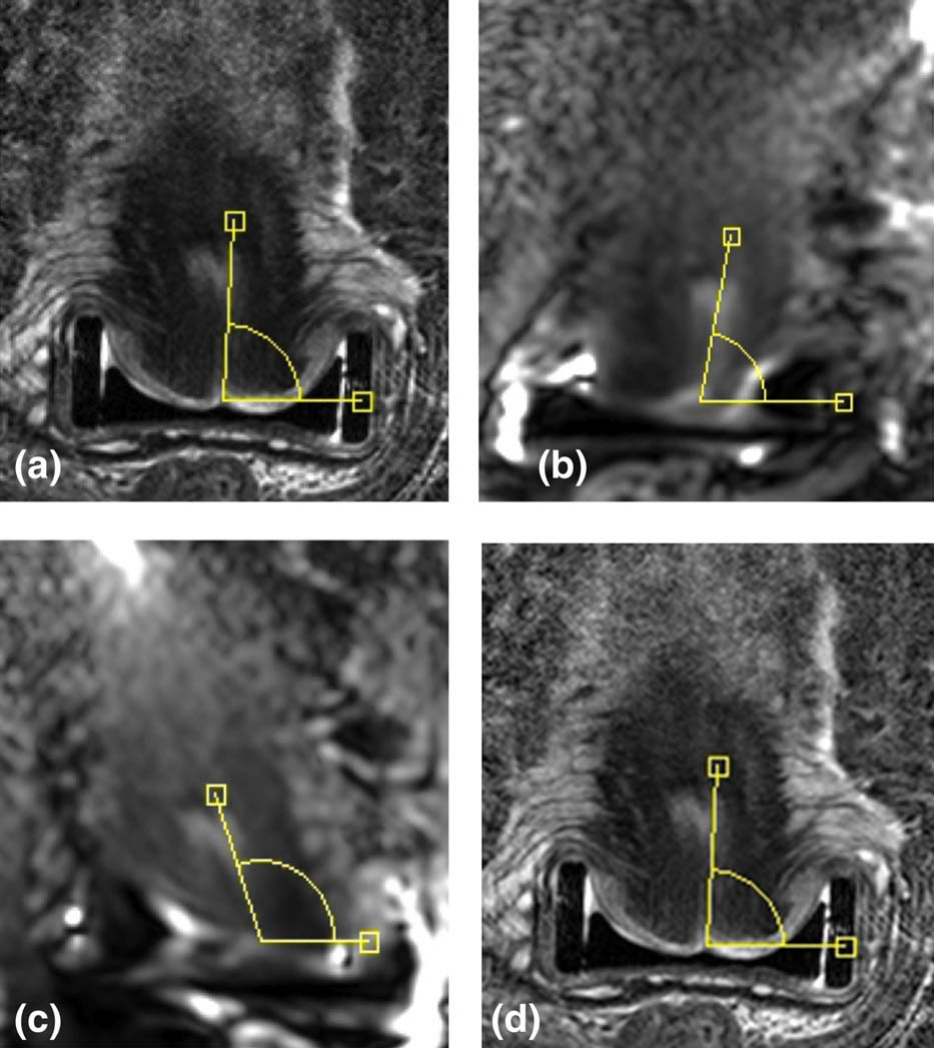
Coronal images through the cervix on *T*_2_W **(a)** with corresponding diffusion‐weighted *b* = 800 s mm^−2^ images acquired using a right gradient **(b)**, left gradient **(c)**, and following correction **(d)**. The angle of the endocervical canal to the x‐axis is also shown: it measures 86° in a, 78° in b, 109° in c, and 86° in d, indicating no difference between the *T*_2_W and corrected diffusion‐weighted images.

The mean angle of the endocervical canal to the x‐axis for all images and the difference in angle measurements between *T*_2_W and diffusion images are given in Table 1. *T*_2_W, right, left, and corrected b = 800 s mm^−2^ images for a coronal slice through the center of the cervix are illustrated in Fig. 1 and the measurement of the angle is shown. The mean angles of the endocervical canal to the x‐axis for the *T*_2_W, right gradient, left gradient, and corrected images were 91.9° ± 11.3°, 85.8° ± 15.8°, 96.7° ± 17.5°, and 91.9° ± 12.1°, respectively. The mean differences in angle of the endocervical canal to the x‐axis between the *T*_2_W, and the right gradient, left gradient and corrected images were 14.8° ± 16.7°, 16.2° ± 20°, and 4.9° ± 6.5°, respectively. Based on the assumption that the *T*_2_W images were ground truth, there was a significant difference between *T*_2_W and right gradient (*P* = 0.007). The difference between *T*_2_W and left gradient approached significance (*P* = 0.055), while there was no difference between *T*_2_W and corrected images (*P* = 0.95). NCC values for all patients over an ROI encompassing the entire cervix on three slices per patient were not significantly different between the uncorrected and the corrected images (0.68 ± 0.15 left gradient, 0.69 ± 0.15 right gradient, 0.68 ± 0.15 corrected images), indicating the insensitivity of the NCC method to track features within the image.

**Table 1:** Angle of the Endocervical Canal to the x‐axis and Difference in Angle of the Endocervical Canal to the x‐axis Between the T2W and the Right Gradient, Left Gradient, and Corrected *b* = 800 s mm^−2^ Images for All Patients

	T2W	Right	Left	Corrected
Angle of endocervical canal to x‐axis (mean ± SD)	91.9˚ ± 11.3˚	85.8˚ ± 15.8˚	96.7˚ ± 17.5˚	91.9˚ ± 12.1˚
Difference angle from T2W (mean ± SD)	NA	14.8˚ ± 16.7˚	16.2˚ ± 20˚	4.9˚ ± 6.5˚

Observer 1 classified 17 of 41 patients as positive for tumor on *T*_2_W+left as well as *T*_2_W+right gradient images and 10 as negative. Corresponding classification for Observer 2 were 19 positive and 8 negative. Two of the former group were endometrial carcinomas. Tumor volume in these patients ranged from 0.07–18.4 cm^3^ (mean ± SD 4.4 ± 5.6 cm^3^). Fourteen of 41 patients (34%) had equivocal findings on *T*_2_W+left as well as *T*_2_W+right gradient images for both observers, 12 of which were common to both observers. Interobserver agreement for classifying images as positive, negative, or uncertain for the presence of tumor before distortion correction was good (kappa = 0.73).

**Figure 2:**
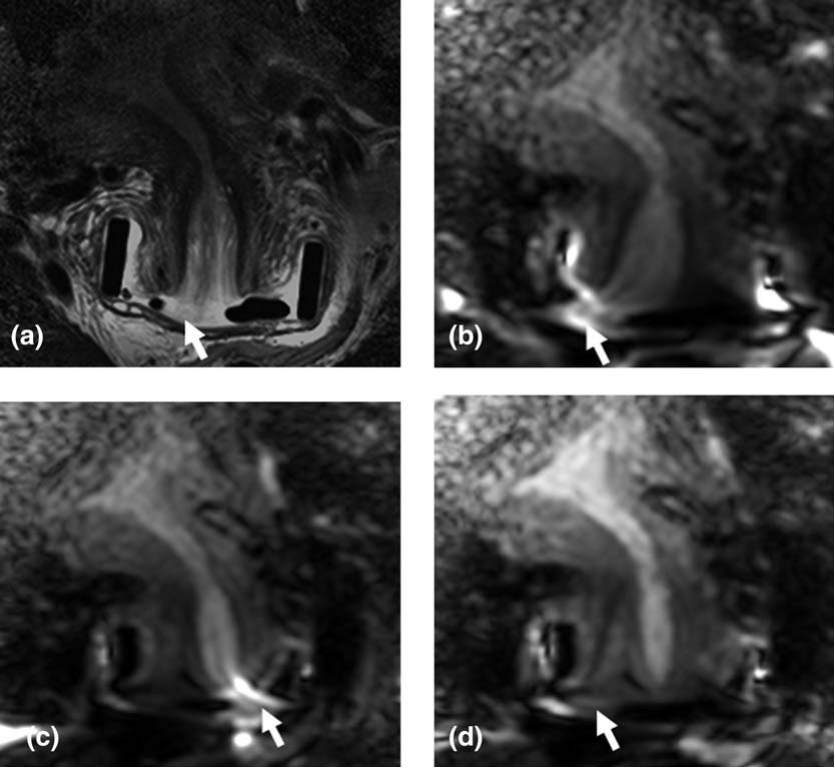
Coronal images through the cervix on *T*_2_W **(a)** with corresponding diffusion‐weighted *b* = 800 s mm^−2^ images acquired using a right gradient **(b)**, left gradient **(c)**, and following correction **(d)**. The intermediate signal intensity nodule of tumor on the right side of the ectocervix in a (arrow) shows diffusion restriction but is displaced and distorted in b and c (arrows). Following correction in d (arrow), it is easier to appreciate its correspondence with the *T*_2_W images.

On viewing the corrected equivocal images with the *T*_2_W images (Fig. 2) Observer 1 classed 11 of 14 cases as positive (tumor volume range 0.01–1.5 cm^3^ (mean ± SD 0.18 ± 0.44 cm^3^) and 3 as negative for tumor, while Observer 2 classified 9 of 14 cases as positive and 5 as negative for tumor. Interobserver agreement for classifying the 12 common uncertain cases as positive or negative for tumor on corrected images was poor (kappa <0.2). Sensitivity and specificity against subsequent histology in this group was 100% (8/8) and 50% (3/6) for Observer 1 and 50% and 20%, respectively, for Observer 2.

## Discussion

We have demonstrated the utility of a phase reversal technique for correcting distortions on EPI DW sequences. The angle of the endocervical canal to the x‐axis indicated that the distortion was greater with the right compared to the left gradient but that on the corrected b = 800 s mm^−2^ images there was a high degree of correlation with the *T*_2_W images. The generation of corrected images therefore allows direct comparison with the *T*_2_W images and should increase the recognition of small cancers while reducing misinterpretation by identification of artifacts. It is notable, however, that the improvement in diagnostic performance of the corrected images was primarily of benefit to an experienced compared to a less‐experienced observer.

Methods of distortion correction on MRI are largely phase‐based^5^ and image‐based techniques,^8^, ^9^, ^10^ all of which were applied to the human brain. Phase‐based techniques yield good corrections but they suffer from increased acquisition time. An example of a phase‐based approach is that of Jezzard and Balaban,^5^ where distortion correction of diffusion images was achieved by acquiring 1D field maps as a function of time in the frequency and phase direction for each slice position and each diffusion gradient strength/direction. The first field map collects a series of complex profiles in the frequency direction from which the frequency‐encode direction eddy current can be calculated. The second field map collects a series of complex profiles in the phase‐encode direction from which the phase‐encode direction eddy current can be calculated. An image‐based technique was suggested by Haselgrove and Moore^10^ utilizing normalized cross‐correlation between phase‐encode lines of a moderate b‐value image (160 s mm^−2^) and a high *b*‐value image. In this approach, distortions were evaluated using a linear least‐square fit to a pair of equations. The cross‐correlation technique was then utilized by Bodammer et al^8^ but image distortions were estimated from a pair of diffusion‐sensitizing gradient images acquired with reversed polarity. This approach was based on the fact that voxel shifts appear in the opposite direction if diffusion‐sensitizing gradients are applied with reversed polarity. Additional low *b*‐value images, as required by Haselgrove and Moore's method, were no longer needed.

For the present implementation, the method of Chang and Fitzpatrick was used^11^ as it offered the simplest solution, albeit requiring an extra image acquisition for the patient. It also had the advantage that the corrected images generated from the combined distorted left and right gradient images were expected to exhibit an SNR higher than either of the distorted images.^6^ Also, despite the limitation that an extra sequence was required, the addition of distortion correction enabled the 34% of equivocal cases with tumor volumes less than 0.2 cm^3^ to be further classified with high sensitivity. The low specificity is likely related to the limits of the spatial resolution of the technique in relation to tumor volume.

Distortions encountered with an EPI‐based DW sequence were particularly pronounced in our study because of the B_1_ field inhomogeneity induced by the endovaginal coil, but the correction methods described here may be applied to any situation or commercial coil where excessive distortion is encountered. Ideally, the correction process requires automation, so that it is implemented immediately following image reconstruction at the scanner. Nevertheless, a limitation is that this correction method incurs a time penalty because of the additional sequence of reversed polarity. Other techniques could have been implemented—turbo spin echo‐based diffusion sequences would be shorter than doing two EPI acquisitions, but are more prone to motion blurring and therefore likely to be diagnostically unhelpful in the assessment of small lesions. Although line‐scan DWI would incur a significant time penalty, it is robust against magnetic susceptibility and bulk motion, and so may be an alternative worth investigating in this clinical application; however, this sequence is not widely available.

The normalized cross‐correlation method for testing for an improvement in agreement with the *T*_2_W images following distortion correction did not prove effective in this study. Contributing factors will include the difference in contrast provided by the two sequences, and the increase in noise in positions distal to the endovaginal RF receiver coil. We did not investigate the effect of the distortion correction on the ADC. It is likely that geometric distortions would affect ADC, not only due to pixel displacement, but also pixel deformation. Miscalculations also result from the fact that images with different diffusion weighting are warped in relation to each other. Furthermore, in these small tumors the number of pixels within a tumor ROI is likely to have been low, further biasing the reported ADC. Therefore, as the objective of the ADC map in these patients was to use the images qualitatively with the *T*_2_W images to identify tumor, quantitation was deemed inappropriate.

Future work will include seeking alternative correction techniques, as the extra acquisition is inconvenient for the patient. The variability of features from one acquisition to the next is also a problem that is particularly evident in slices closest to the bladder, despite the fact that patients emptied their bladder immediately prior to the examination. One could also attempt to register the DW images to their *T*_2_W counterpart using an affine‐based approach. Correcting distortion in DW images particularly at field strengths of 3T and above remains a challenging problem due to increased magnetic field inhomogeneity and this is still an area of active research.

In conclusion, application of the reverse gradient algorithm for distortion correction of echo‐planar DWI was implemented and improved correspondence with *T*_2_W images. The diagnostic performance was improved in a third of cases for the more experienced observer.
